# Performance of the Four-Plex Tandem Mass Spectrometry Lysosomal Storage Disease Newborn Screening Test: The Necessity of Adding a 2nd Tier Test for Pompe Disease

**DOI:** 10.3390/ijns4040041

**Published:** 2018-12-18

**Authors:** Shu-Chuan Chiang, Pin-Wen Chen, Wuh-Liang Hwu, An-Ju Lee, Li-Chu Chen, Ni-Chung Lee, Li-Yan Chiou, Yin-Hsiu Chien

**Affiliations:** 1 Department of Medical Genetics, National Taiwan University Hospital, Taipei 10041, Taiwan; 2 Department of Pediatrics, National Taiwan University College of Medicine, Taipei 10041, Taiwan; 3 Department of Pediatrics, National Taiwan University Children Hospital, Taipei 10041, Taiwan

**Keywords:** Pompe newborn screening, tandem mass spectrometry, Gaucher newborn screening, Fabry newborn screening, accuracy

## Abstract

Early diagnosis of lysosomal storage diseases (LSDs) through newborn screening (NBS) has been adapted widely. The National Taiwan University Hospital Newborn Screening Center launched the four-plex tandem mass spectrometry LSD newborn screening test in 2015. The test determined activities of acid α-glucosidase (GAA; Pompe), acid α-galactosidase (GLA; Fabry), acid β-glucocerebrosidase (ABG; Gaucher), and acid α-l-iduronidase (IDUA; MPS-I) in dried blood spots (DBS). Through 2017, 64,148 newborns were screened for these four LSDs. The screening algorithm includes enzyme activity/ratio as the cutoffs for the first screening test and a second-tier test for Pompe disease screening. The second-tier Pompe disease screening test measured activity inhibition by acarbose. Twenty-nine newborns required a confirmatory test; six were confirmed to have Pompe disease, and nine were confirmed to have Fabry disease. The screen-positive rate for Pompe disease was 0.031%. Therefore, in Pompe disease newborn screening, a validated 2nd tier test is necessary to decrease false positives.

## 1. Introduction

Lysosomal storage diseases (LSDs) are caused by a deficiency of one of the lysosomal acid hydrolases. Currently, many LSDs are treatable with enzyme replacement, pharmaceutical chaperones, substrate reduction therapy, and/or bone marrow transplantation. Because LSDs can lead to irreversible damage to the tissues and organs of affected children, screening newborns for treatable LSDs has been conducted in different populations with the goal of reducing disease-related morbidity and mortality through early treatment.

Pompe disease (also known as glycogen storage disease type II, glycogenosis II, or acid maltase deficiency) is a lysosomal storage disorder in which a deficiency in acid α-glucosidase (GAA) causes the intralysosomal accumulation of glycogen in all tissues, most notably skeletal muscles [1]. Through newborn screening, patients with the severe infantile-onset Pompe disease (IOPD) have been shown to benefit from early diagnosis and early treatment [2,3]. Therefore, in March 2015, the US Secretary of Health and Human Services approved the recommendation made in May 2013 by the Advisory Committee on Heritable Disorders in Newborns and Children (ACHDNC) to add Pompe disease to the Recommended Uniform Screening Panel (RUSP). However, the first large-scale newborn screening for Pompe disease [2,4] had a recall rate of 0.82% [4]. After we realized the presence of a common pseudodeficiency allele in the Taiwanese population, we improved the screening algorithm by adding a 2nd tier test measuring neutral α-glucosidase (NAG) and total α-glucosidase (TAG) activities. This approach reduces the false positive rate to a recall rate of lower than 0.26% [5].

Currently, newborn screening has been performed for a panel of LSDs, and therefore, multiplex LSD newborn screening assays have also been developed. These assays are either based on measuring LSD enzyme activities using a tandem mass spectrometry (MS/MS) substrates or using the digital microfluidic fluorometric method. Since 2015, we shifted our fluorimetric newborn screening assays for Pompe to a four-plex MS/MS assay for Pompe disease, mucopolysaccharidosis type I (MPS I), Gaucher disease and Fabry disease. Given the presence of pseudodeficiency in the Taiwanese population, a second-tier test for Pompe disease was still added to the multiplex test. We report our results from screening of the first 60,000 newborns using the MS/MS method to demonstrate the necessity of a 2nd tier test for Pompe disease.

## 2. Materials and Methods

### 2.1. Study Population

The Newborn Screening Center at the National Taiwan University Hospital (NTUH) initiated a pilot screening program for Pompe disease in 2005. The center performs routine newborn screening for approximately 35% of newborns in Taiwan, or 70,000 newborns per year. Pompe disease screening was added to the regular screening items in 2008. In 2015, we started the four-plex assay: Pompe disease, MPS I, Gaucher disease, and Fabry disease. Parents of newborns needed to give consent for the LSD multiplex assay. The LSD multiplex assay used the residual dried blood spots (DBS) specimens collected for routine NBS, so no additional blood sampling was required.

### 2.2. Screening Assay

DBS GAA activity was previously measured using 4-methylumbelliferyl-α-d-glucopyranoside (4-MU-glucoside) as a substrate [4] (4MU assay). The MS/MS LSD multiplex assay was used since 2015 due to its multiplex ability. The MS/MS substrates and internal standards were provided through a collaboration between Genzyme Pharmaceuticals and the Centers for Disease Control and Prevention (CDC assay) until November 2017, and PerkinElmer (PE NeoLSD) provided the substrates and internal standards after that. The MS/MS substrates and internal standards for neutral α-glucosidase (NAG) were made and provided by Michael Gelb from University of Washington, Seattle, USA.

In our previous 4MU assay and the later CDC MS/MS multiplex assay, both the first screening cutoff and the critical cutoff for Pompe disease was based on the NAG/GAA ratio, but the cutoffs were changed to acid β-glucocerebrosidase (ABG)/GAA ratio in the MS/MS multiplex assay in 2016. Newborns with a screening result exceeding the critical screening cutoff (ABG/GAA ≥ 20) are subjected to confirmation immediately without doing the second tier assay. Newborns with a screening result exceeding the first cutoff (ABG/GAA ≥ 8 or GAA ≤ 1.2 µM/h) but not reaching the critical cutoff proceed to the second tier assay. The second tier assay was still based on 4MU substrates, calculating the percent inhibition by acarbose [5]. Briefly, two assays were performed: (1) GAA activity, measured at pH 3.8 in the presence of acarbose; and (2), total GAA (tGAA), measured at pH 3.8 without acarbose. The percent inhibition by acarbose was calculated with the formula: (tGAA-GAA)/tGAA, and if the value was equal or over 87%, the newborns were regarded as screening-positive and the confirmation process were followed.

The cutoff for Fabry disease was set at 15% of normal mean acid α-galactosidase (GLA) activity, and targeted approximately 1 per 10,000 newborns. To further avoid detection of type II Fabry disease (the later-onset type) [6], we set the cutoff for Fabry disease based on the ABG/GLA ratio. For Gaucher disease and MPS I, the cutoffs were 20% and 30% enzyme activity of the population mean, respectively. For newborns who had first screening results exceeding the cutoffs in these three diseases, enzyme activities were tested again (retest) on a new punch from the original dried blood filter. Newborns with an abnormal retest result have their 2nd sample tested, and then proceed through confirmatory testing if the 2nd sample still shows an abnormal result.

### 2.3. Confirmatory Testing

Newborns were referred to NTUH for confirmatory testing. The tests included lymphocyte or leukocyte enzyme activity measurement, mutation analysis and biomarker measurement [2,4,7]. Clinical diagnosis and phenotypic prediction were based on the results of confirmatory testing and clinical findings. Predicted early-onset phenotypes included classic infantile-onset Pompe disease, types 2 or 3 Gaucher disease, type 1 Fabry disease, and severe type of MPS 1. Predicted late-onset phenotypes included late-onset Pompe disease, type 1 Gaucher disease, type 2 Fabry disease, and attenuated MPS 1.

## 3. Results

Between Jan and Oct 2017, 64,147 newborns were tested by the four-plex MS/MS assay. Fifty-seven samples were regarded as unsatisfactory due to low levels of all four enzyme activities, and they were requested to submit a second sample. Overall referral numbers and results of confirmatory testing are summarized in Table 1. Eighty-five (0.1%) infants were screen-positives at the 1st sample. Of these, 29, or 34%, were still screen-positive after a repeat newborn screen or, in the case of Pompe, application of the second-tier test and required further confirmatory testing. Fifteen of the 29 referred infants were determined to be patients, including two early-onset patients. The other 13 patients were determined to be at risk for late-onset disease; all of these infants had genotypes known to be associated with late-onset phenotypes or unknown significance, low enzyme activity, but none had relevant clinical symptoms at the most recent evaluation. The remaining 14 (48% of referrals) were false positives with normal confirmatory results.

**Figure 1:**
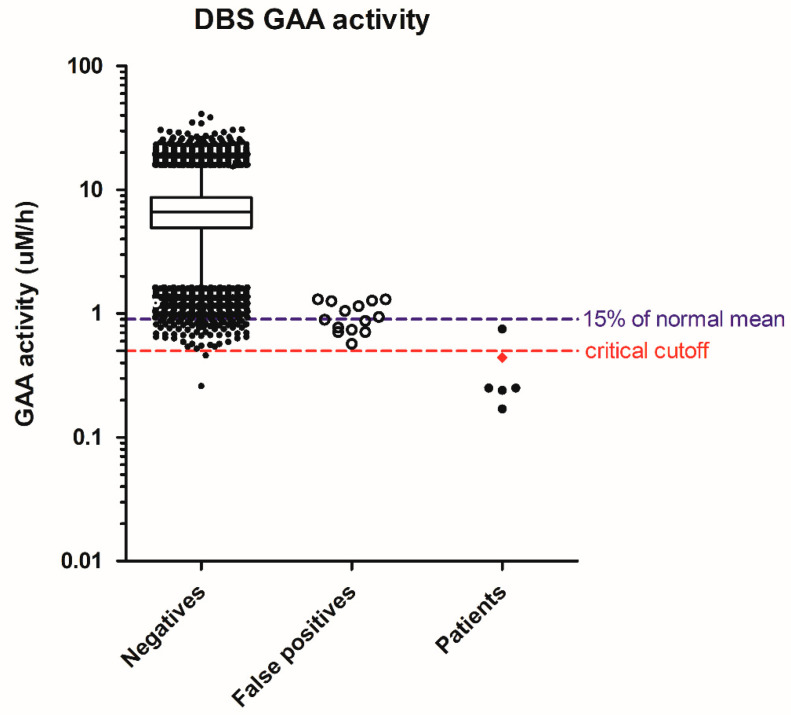
Distribution of screening acid α-glucosidase (GAA) activity in negatives, false positives, and patients. Whiskers: 1–99th percentile; box: 25–75th percentile; line in box: median; diamond in patients: IOPD; solid dots in patients: suspect LOPD.

In Pompe disease screening, 64,148 specimens were screened, resulting in 20 referrals for a screen-positive rate of 0.031%. There were six infants with deficiency of lymphocyte GAA enzyme activity and two *in trans GAA* pathologic mutations, one with cardiomegaly at birth and receiving treatment immediately. This IOPD patient had the initial screening GAA activity in DBS as 0.44 uM/h, or 6% of the normal mean. The patients suspected of having later-onset PD (LOPD) had a GAA activity range of 0.17 to 0.75 µM/h, or 2% to 11% of the normal mean. The GAA activity in false positives ranged from 0.57 to 1.3 µM/h, or 8% to 19% of the normal mean. The highest GAA activity in the false positives equaled 0.5 percentile of the population. We have since strengthened the cutoffs to the 0.1 percentile of the population as 0.85 uM/h, or 13–15% of the normal mean. In that case, we would have been able to eliminate seven false positives and improve the positive prediction rate from 30% to 46% in this cohort. However, without our 2nd tier test, the single GAA assay would result in an additional 72 infants having recall or confirmatory tests, i.e., 1 out of the 1000 newborns (Figure 1). In addition, the critical cutoff using GAA activity may be set at 0.5 µM/h, but the laboratories need to be aware of test variation, and the confirmatory process for our IOPD patient (diamond, Figure 1) would have been delayed by chance.

**Figure 2:**
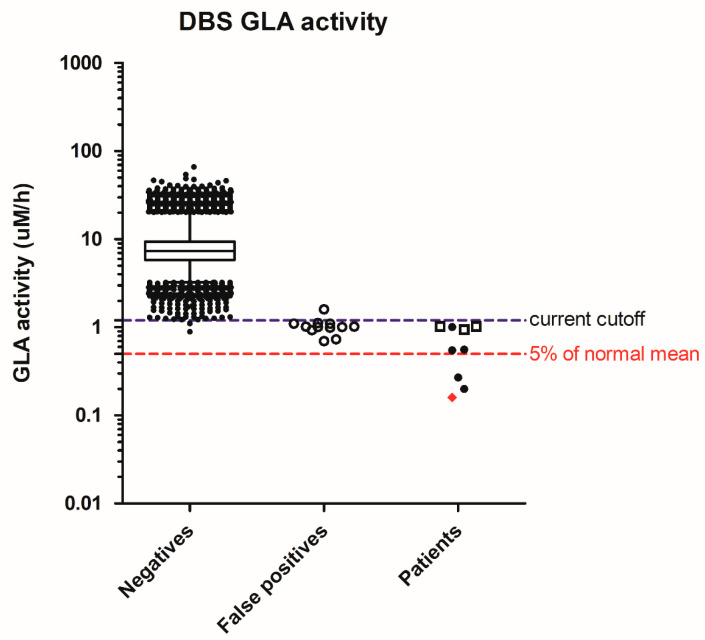
Distribution of screening acid α-galactosidase (GLA) activity in negatives, false positives, and patients. Whiskers: 1–99th percentile; box: 25–75th percentile; line in box: median; Diamond in patients: classic mutation; solid dots in patients: suspect type 1 mutation or confirm type 2 mutations; hollow squares in patients: c.636 + 919G>A mutation.

In Fabry disease screening, 64,148 specimens were screened, resulting in 21 abnormal samples for a 2nd DBS. Finally, nine need a referral, and the screen-positive rate was 0.014% (Table 2). There were nine males with low enzyme activity, but only 1 had type 1, classical Fabry mutation, and his GLA activity was 2% of the normal mean. Three of the remaining eight males had the common c.636 + 919G>A (also known as IVS4 + 919G>A) on the *GLA* gene that causes the late-onset cardiac phenotype, and their DBS GLA activities were above 10% of the normal mean. To further decrease the chance of detecting the later-onset cardiac phenotypes, it is reasonable to further strengthen the cutoffs to 0.5 µM/h (Figure 2), or 5% of the normal mean, equal to 0.008 percentile of the population.

In MPS I and Gaucher disease screening, 34 and 10 newborns were requested to test the 2nd DBS, respectively. All revealed normal enzyme activities thereafter. Therefore, the incidences for both diseases are below 1 in 60,000 newborns. Combining these results with our previous year’s result, the incidence of MPS I and Gaucher in our screened population is 2 and 1 in ~150,000 newborns, respectively.

## 4. Discussion

Our results confirm the importance of a suitable 2nd tier test in Pompe screening. As the first pilot screening program in the world, we intended to include more infants for further testing, and therefore, the recall rate was 0.8% initially [4]. Infants with Pompe and MPS1 pseudodeficiency alleles [8,9] were screen-positives. Using a reliable second-tier test, referring such cases for confirmatory testing could be prevented. However, in Pompe disease, we have shown that complete molecular analysis is needed if using as a 2nd tier test [8], because the pseudodeficiency allele may exist alone or combine with a pathologic mutation. Therefore, we have developed a ratiometric assay to exclude pseudodeficiency newborns from referrals [5]. Transforming the screening assay from a manual fluorometric method to microfluidic or MS/MS partially solves but does not eliminate the problem [9,10,11,12,13]. In addition, those studies were conducted in populations different from ours, where the pseudodeficiency allele is prevalent in Asians. Another study using the MS/MS method in a similar population claims the superiority of MS/MS, but it was a method validation study and may be biased by its retrospective study design [14]. Different approaches on top of the MS/MS method, such as the use of second-tier biochemical test using the ratio of creatine/creatinine [15] and the Mayo Clinic Collaborative Laboratory Integrated Reports (CLIR) tool [16], may help in the accuracy but have not been tested in different populations. In our current study, we demonstrate our ratiometric approach (either by the fluorometric substrate or the MS/MS substrate) is reliable and can safely distinguish the Pompe-affected infants from pseudodeficiency without sacrificing the efficiency of timely treatment.

In our program, we only aimed to detect newborns with classic Fabry disease but not newborns with the risk of type 2 late-onset Fabry disease. The reasons are clear: it may take decades to determine whether these individuals express the disease phenotype, and the pressure on the babies and the parents is enormous and may last for a long time. Since the cardiac type variant is too prevalent in our population, found in up to 1 in 800 in males [6], and we can modify the cutoffs to detect only the most severe type patients, we therefore set a very strict cutoff to detect only infants with enzyme activity less than 5% of the normal mean. Further phenotypic descriptions are imperative in helping us to determine cutoff and screening algorithms. Our approach should be able to detect females with the most severe classic Fabry disease since we don’t apply the sex in the decision tree. However, there was no such case during this period.

The disease incidence in different populations is difficult to compare due to the effect of populations, the definition of late-onset type, and the methods and cutoffs applied. For example, the incidence of Pompe disease ranges from 1 in 14,567 in Missouri [10] to 1 in 21,979 in Illinois [11], similar to our incidence of 1 in 11,000 in the current study. The incidence of Gaucher disease, however, is high in New York (1 in 4374) [9] and other programs (1 in 43,000) [10,11] but is low in our program (1 in 150,000). Because we also use the referred cutoffs as 20% of the normal mean, the best explanation is a genetic difference but not the definition of a “late-onset” type or the sensitivity of the assays to detect such kind of newborns.

## 5. Conclusions

In summary, with our long experience in Pompe newborn screening, we demonstrate that a suitable 2nd tier test is still necessary to decrease the false positive rate and facilitate the referral process for true positives. We also demonstrate that it is possible to decrease the chance of identifying newborns with the risk of type 2 Fabry disease because they tend to have higher residual enzyme activity. Further knowledge and discussion focusing on the evidence and the ethical and practical issues will be essential to decide whether identifying newborns at risk for late-onset disease through the screening is valuable. Future pilot NBS studies may utilize this infrastructure and the multiplex format to obtain relevant information on other treatable LSD diseases.

## Figures and Tables

**Table 1:** Overall screening results.

LSD	Repeat NBS (on 2nd DBS)	False Positives	Patients	Early Onset	Later-Onset
Pompe	-	14	6	1	5
Fabry	21	0	9	1	8
MPS I	34	0	0	0	0
Gaucher	10	0	0	0	0

**Table 2:** NBS and confirmatory testing results of referred infants, and diagnostic determination.

Disorders Suspected	GAA	GLA	ABG	ABG/GAA	ABG/GLA	Inhibition (%) ^#^	% of mean DBS Activity	WBC Activity *	Diagnosis
**Pompe**	0.44	11.0	22.1	50.4	2.00	-	6%	0.11	IOPD
**Pompe**	0.17	8.62	15.3	88.4	1.77	-	2%	0.93	LOPD
**Pompe**	0.24	8.16	8.16	33.7	1.00	-	3%	0.74	LOPD
**Pompe**	0.25	11.1	19.8	77.9	1.78	-	4%	0.50	LOPD
**Pompe**	0.25	8.29	10.2	40.0	1.23	-	4%	0.30	LOPD
**Pompe**	0.75	11.5	13.6	18.1	1.18	88.0	13%	0.76	LOPD
**Pompe**	0.57	3.63	4.72	8.28	1.30	87.0	8%	5.56	Not affected
**Pompe**	0.71	9.87	16.8	23.6	1.70	-	10%	4.43	Not affected
**Pompe**	0.71	11.7	14.5	20.3	1.23	-	10%	1.16	Not affected
**Pompe**	0.74	5.93	9.97	13.5	1.68	89.5	11%	4.07	Not affected
**Pompe**	0.77	6.60	11.7	15.2	1.78	87.4	11%	3.58	Not affected
**Pompe**	0.87	3.32	11.1	12.8	3.34	87.7	12%	2.11	Not affected
**Pompe**	0.89	5.83	9.10	10.2	1.56	89.0	13%	3.54	Not affected
**Pompe**	0.94	3.67	11.7	12.4	3.20	87.1	13%	3.86	Not affected
**Pompe**	1.05	4.69	8.49	8.06	1.81	87.8	15%	-	Not affected
**Pompe**	1.15	5.11	8.59	7.45	1.68	87.4	16%	4.08	Not affected
**Pompe**	1.26	3.47	10.5	8.34	3.02	87.5	18%	3.98	Not affected
**Pompe**	1.27	8.74	14.6	11.5	1.67	88.3	18%	-	Not affected
**Pompe**	1.30	7.78	12.0	9.26	1.55	87.2	19%	4.56	Not affected
**Pompe**	1.30	6.74	17.9	13.8	2.66	87.3	19%	3.91	Not affected
**Fabry**	3.57	0.16	7.26	2.03	44.1	-	2%	0.42	T1FD
**Fabry**	8.20	0.27	9.05	1.10	33.9	-	3%	1.00	(S) T1FD
**Fabry**	5.10	0.20	16.8	3.29	85.9	-	2%	0.88	T2FD
**Fabry**	2.42	0.55	6.89	2.85	12.6	-	7%	4.62	(S) T2FD
**Fabry**	6.32	0.56	16.4	2.60	29.4	-	7%	-	(S) T2FD
**Fabry**	6.85	1.02	9.09	1.33	8.89	-	13%	4.53	IVS4 + 919A
**Fabry**	5.64	1.02	14.5	2.57	15.0	-	13%	3.01	IVS4 + 919A
**Fabry**	10.5	1.01	9.22	0.88	9.14	-	13%	6.50	IVS4 + 919A
**Fabry**	8.86	1.01	13.3	1.50	15.0	-	13%	6.24	Benign

**^#^** The cutoff for the inhibition (%) was 87. In Pompe disease, only cases met the screening criteria but not the critical criteria would be tested for the inhibition (%). Cases met the critical criteria (ABG/GAA ≥ 20) need the confirmation testings immediately and therefore no values for this inhibition (%). *: Lymphocyte GAA activity with the presence of inhibitor (Normal mean = 25) in newborns suspected of having Pompe disease, leukocyte GLA activity (Normal mean = 60) in newborns suspected of having Fabry disease. GAA: acid α-glucosidase; GLA: acid α-galactosidase; ABG: acid β-glucocerebrosidase; inhibition: % of GAA activity inhibited by acarbose; DBS: dried blood spots; IOPD: infantile-onset Pompe disease; LOPD: later-onset Pompe disease; (S): suspect; T1FD: classical or type 1 Fabry disease; T2FD: non-classical or type 2 Fabry disease. -: Not done.

## References

[1] Hirschhorn R., Reuser A., Scriver C., Beaudet A., Sly W., Valle D. (2001). Glycogen storage disease type II: Acid α-glucosidase (acid maltase) deficiency. The Metabolic and Molecular Bases of Inherited Disease.

[2] Hwu W.L., Chien Y.H., Lee N.C., Chiang S.C., Dobrovolny R., Huang A.C., Yeh H.Y., Chao M.C., Lin S.J., Kitagawa T. (2009). Newborn screening for fabry disease in taiwan reveals a high incidence of the later-onset *GLA* mutation c.936+919G>A (IVS4+919G>A). Hum. Mutat..

[3] Chien Y.H., Lee N.C., Chen C.A., Tsai F.J., Tsai W.H., Shieh J.Y., Huang H.J., Hsu W.C., Tsai T.H., Hwu W.L. (2015). Long-term prognosis of patients with infantile-onset pompe disease diagnosed by newborn screening and treated since birth. J. Pediatr..

[4] Chien Y.H., Chiang S.C., Zhang X.K., Keutzer J., Lee N.C., Huang A.C., Chen C.A., Wu M.H., Huang P.H., Tsai F.J. (2008). Early detection of pompe disease by newborn screening is feasible: Results from the taiwan screening program. Pediatrics.

[5] Chiang S.C., Hwu W.L., Lee N.C., Hsu L.W., Chien Y.H. (2012). Algorithm for pompe disease newborn screening: Results from the taiwan screening program. Mol. Genet. Metab..

[6] Chien Y.H., Lee N.C., Chiang S.C., Desnick R.J., Hwu W.L. (2012). Fabry disease: Incidence of the common later-onset α-galactosidase a IVS4+919G>A mutation in taiwanese newborns—Superiority of DNA-based to enzyme-based newborn screening for common mutations. Mol. Med..

[7] Chien Y.H., Lee N.C., Thurberg B.L., Chiang S.C., Zhang X.K., Keutzer J., Huang A.C., Wu M.H., Huang P.H., Tsai F.J. (2009). Pompe disease in infants: Improving the prognosis by newborn screening and early treatment. Pediatrics.

[8] Labrousse P., Chien Y.H., Pomponio R.J., Keutzer J., Lee N.C., Akmaev V.R., Scholl T., Hwu W.L. (2010). Genetic heterozygosity and pseudodeficiency in the Pompe disease newborn screening pilot program. Mol. Genet. Metab..

[9] Wasserstein M.P., Caggana M., Bailey S.M., Desnick R.J., Edelmann L., Estrella L., Holzman I., Kelly N.R., Kornreich R., Kupchik S.G. (2018). The New York pilot newborn screening program for lysosomal storage diseases: Report of the first 65,000 infants. Genet. Med..

[10] Hopkins P.V., Campbell C., Klug T., Rogers S., Raburn-Miller J., Kiesling J. (2015). Lysosomal storage disorder screening implementation: Findings from the first six months of full population pilot testing in missouri. J. Pediatr..

[11] Burton B.K., Charrow J., Hoganson G.E., Waggoner D., Tinkle B., Braddock S.R., Schneider M., Grange D.K., Nash C., Shryock H. (2017). Newborn screening for lysosomal storage disorders in illinois: The initial 15-month experience. J. Pediatr..

[12] Elliott S., Buroker N., Cournoyer J.J., Potier A.M., Trometer J.D., Elbin C., Schermer M.J., Kantola J., Boyce A., Turecek F. (2016). Dataset and standard operating procedure for newborn screening of six lysosomal storage diseases: By tandem mass spectrometry. Data Brief.

[13] Burlina A.B., Polo G., Salviati L., Duro G., Zizzo C., Dardis A., Bembi B., Cazzorla C., Rubert L., Zordan R. (2018). Newborn screening for lysosomal storage disorders by tandem mass spectrometry in north east italy. J. Inherit. Metab. Dis..

[14] Liao H.C., Chan M.J., Yang C.F., Chiang C.C., Niu D.M., Huang C.K., Gelb M.H. (2017). Mass spectrometry but not fluorimetry distinguishes affected and pseudodeficiency patients in newborn screening for pompe disease. Clin. Chem..

[15] Tortorelli S., Eckerman J.S., Orsini J.J., Stevens C., Hart J., Hall P.L., Alexander J.J., Gavrilov D., Oglesbee D., Raymond K. (2017). Moonlighting newborn screening markers: The incidental discovery of a second-tier test for pompe disease. Genet. Med..

[16] Minter Baerg M.M., Stoway S.D., Hart J., Mott L., Peck D.S., Nett S.L., Eckerman J.S., Lacey J.M., Turgeon C.T., Gavrilov D. (2017). Precision newborn screening for lysosomal disorders. Genet. Med..

